# Vitexin ameliorates glycochenodeoxycholate-induced hepatocyte injury through SIRT6 and JAK2/STAT3 pathways

**DOI:** 10.22038/IJBMS.2021.59424.13196

**Published:** 2021-12

**Authors:** Chuang Zhang, Suolin Li, Chi Sun, Lin Liu, Yanbin Fang, Xiaofeng Yang, Xingxin Pan, Ben Zhang

**Affiliations:** 1 Department of Pediatric Surgery, Second Hospital of Hebei Medical University, Shijiazhuang, Hebei, 050000, China

**Keywords:** Apoptosis, Cholestasis, Glycochenodeoxycholic acid, Necroptosis, Oxidative stress, SIRT6, Vitexin

## Abstract

**Objective(s):**

Vitexin, a natural flavonoid, is commonly found in many foods and traditional herbal medicines and has clear health benefits. However, the role of vitexin in cholestasis is presently unclear. This study investigated whether vitexin mitigated glycochenodeoxycholate (GCDC)-induced hepatocyte injury and further elucidated the underlying mechanisms.

**Materials and Methods:**

A cell counting kit-8 (CCK-8) assay was conducted to evaluate cell viability. The mitochondrial membrane potential (MMP, Δψm), reactive oxygen species (ROS) levels, and apoptosis rate of hepatocytes exposed to GCDC were detected by flow cytometry (FCM). We then measured the cytoprotective effects of vitexin against oxidative stress. The molecular signaling pathway was further investigated by using Western blotting and signaling pathway inhibitors.

**Results:**

Here, we showed that vitexin increased cell viability and reduced cell apoptosis, necroptosis, and oxidative stress in a dose-dependent manner in GCDC-treated hepatocytes. In addition, by using selective inhibitors, we further confirmed that inhibition of the JAK2/STAT3 pathway by vitexin was mediated by prolonged activation of Sirtuin 6 (SIRT6).

**Conclusion:**

Vitexin attenuated GCDC-induced hepatocyte injury via SIRT6 and the JAK2/STAT3 pathways.

## Introduction

Cholestasis, one of the most common conditions of the liver in childhood, is characterized by the accumulation of toxic bile acids (BAs) in the liver and systemic circulation, which causes severe liver dysfunction and, ultimately, cirrhosis and premature death (1, 2). 

The hydrophobic BA GCDC is the main component of human serum and bile during cholestasis. Previous evidence has indicated that toxic BAs can induce hepatocyte apoptosis (3). Recent studies, however, have demonstrated that necroptosis might be the dominant type of cell death in rodent models of cholestasis. Receptor-interacting protein kinase (RIPK)1 and RIPK3 are phosphorylated in the necrosome and activated RIPK3 phosphorylates the mixed lineage kinase domain-like (MLKL) protein, inducing it to self-oligomerize and execute necrosis (4, 5). Both apoptosis and necroptosis are likely to occur in cholestatic diseases. Nevertheless, both apoptosis and necrosis involve mitochondria. Mitochondrial dysfunction and overproduction of ROS play key roles in the progression of liver disease (6, 7). Hydrophobic BAs can stimulate ROS production as well as oxidative stress, and superoxide dismutase (SOD), glutathione peroxidase (GSH-Px), and malondialdehyde (MDA) are common indicators of oxidative stress (8). ROS can depolarize MMP, which eventually triggers mitochondrial-dependent apoptosis. In the mitochondrial-dependent apoptosis pathway, B-cell lymphoma 2 (Bcl-2) is an antiapoptotic protein, Bcl2-associated X protein (BAX) is a proapoptotic protein, and caspase-3 is the major executor of apoptosis (9, 10). 

SIRT6 is an NAD+-dependent histone deacetylase that belongs to the Sirtuin family. Research has revealed that sirtuins play a crucial regulatory role in the pathophysiological processes of different types of liver injury, such as fatty liver, hepatitis, hepatic fibrosis, and liver cancer (11-13). The JAK2/STAT3 pathway mediates numerous cytokine signaling pathways and participates ubiquitously in the processes of cell proliferation, differentiation, apoptosis, and inflammation. The JAK2/STAT3 pathway is also activated in liver tissues, and abnormal signal transduction can contribute to multiple liver injuries (14-16). However, the role of SIRT6 and JAK2/STAT3 pathway in GCDC-induced liver damage has not previously been reported.

Vitexin is a plant-derived natural flavonoid found in various edible and medicinal plants. It is commonly used for anti-inflammatory, antibacterial, anti-oxidant, and antitumor activities (17). Nevertheless, there is still a lack of information concerning the effect of vitexin on cholestatic liver diseases (CLDs). Currently, only two clinical drugs, ursodeoxycholic acid (UDCA) and obeticholic acid (OCA) have been approved for cholestasis treatment by the US Food and Drug Administration (FDA), but their treatment effects are limited. There is an urgent need to find novel therapeutic agents for cholestasis (18). 

Therefore, the purpose of this study is to investigate whether vitexin is effective in treating GCDC-induced hepatocyte injury and analyze it in terms of oxidative stress, MMPs, apoptosis, and necroptosis. There are comparisons to UDCA. Moreover, OSS_128167, an inhibitor of SIRT6, was used in this study to further investigate the relationship between SIRT6 and JAK2/STAT3 pathways, and their functions in the hepatoprotective effects of vitexin.

## Materials and Methods

***Materials***

Human normal hepatocyte THLE-3 cells were obtained from the Shanghai Cell Bank of the Chinese Academy of Sciences (China). Vitexin (≥ 98%) was purchased from Chengdu Pufei De Biotech Co., Ltd. (China). UDCA (99%) was obtained from Aladdin (China). GCDC was purchased from Sigma-Aldrich (USA). OSS_128167 was provided by MedChemExpress (USA)**. **RPMI-1640 medium, fetal bovine serum (FBS), and 1% (v/v) penicillin-streptomycin solution were obtained from Gibco (USA). CCK8 was obtained from Dojindo (Japan). The apoptosis assay kit was purchased from Absin (China). The MMP detection kit (JC-10) and ROS detection kit were provided by KeyGEN (China). SOD, GSH, and MDA assay kits were from Suzhou Geruisi Biotechnology Co., Ltd. (China). Radioimmunoprecipitation assay (RIPA) buffer with 1% protease and phosphatase inhibitor and a BCA kit were obtained from Solarbio (China). Primary antibodies were purchased from Cell Signaling Technology (USA) (cleaved caspase-3 (cleaved-casp3), p-JAK2, JAK2, p-STAT3, STAT3, SIRT6, p-RIPK1, p-RIPK3, and p-MLKL) and Bioworld Biotechnology (China) (Bcl-2, Bax and GAPDH).

***Cells and treatments***

THLE-3 cells were cultured in RPMI-1640 medium with 10% FBS and 1% (v/v) penicillin-streptomycin solution at 37 °C and 5% CO_2_. The cells were processed in the following manner: GCDC groups were stimulated with GCDC (200 μM) alone for 24 hr (19, 20); UDCA treatment groups were preincubated with UDCA (100 μM) for 2 hr before GCDC stimulation (21); vitexin treatment groups were preincubated with vitexin (5 μM/10 μM/20 μM) for 2 hr before GCDC stimulation; OSS_128167 groups were first treated with OSS_128167 (100 μM) for 30 min and then preincubated with vitexin (20 μM) for 2 hr before GCDC stimulation (22).

***Cell viability assay***

THLE-3 cells were seeded in a 96-well plate at a density of 1×10^4 ^cells/well in the presence of vitexin at different concentrations (0 μM, 5 μM, 10 μM, and 20 μM) for 24 hr. THLE-3 cells were seeded in a 96-well plate at a density of 1×10^4 ^cells/well in the presence of vitexin at different concentrations (0 μM, 5 μM, 10 μM, and20 μM) for 2 hr, then GCDC was added at a concentration of 200 μM, followed by incubation for another 24 hr. CCK-8 was added to cells and incubated for 4 hr, and the viability of the cells was measured at 450 nm using a multifunctional microplate reader (SPARK 10M, TECAN, Switzerland).

***MMP measurement***

Cells were grouped and treated as described before, collected, and resuspended in 0.5 ml incubation buffer containing 10 μg/ml JC-10 (enhanced JC-1). Then, the cells were incubated at 37°C with 5% CO_2_ for 20 min and further analyzed by FCM.

***ROS assay***

The intracellular ROS levels were measured using an ROS detection kit. After grouping and treatment as described above, THLE-3 cells were incubated with DCFH-DA (10 μM) in the dark at 37 °C for 60 min. Then, the cells were washed three times with PBS, and ROS production was measured by FCM following the manufacturer’s instructions.

***SOD, GSH, and MDA measurement***

Cells were grouped and treated as described before, collected, and homogenized. The contents of SOD, GSH, and MDA in the homogenate were determined according to the kit instructions.

***Apoptosis analysis***

The percentage of apoptotic THLE-3 cells was measured using a commercial kit. Cells were grouped and treated as described before and stained with FITC-labelled Annexin V (1 mg/ml) and propidium iodide (10 mg/ml) for 15 min. After gentle washes, patterns of apoptotic cells were measured using a FACS Aria II (BD, FACS Aria II, USA).

***Western blots***

Cells were lysed with RIPA buffer. The concentration of total protein was measured with a BCA kit. A few of samples were separated on SDS-PAGE gels and transferred onto 0.22 µM PVDF membranes. The membranes were blocked with 5% BSA-PBST buffer for 1 hr, followed by incubation with primary antibodies against Bcl-2 (1:500), Bax (1:500), cleaved caspase-3 (1:1000), p-RIPK1 (1:1000), p-RIPK3 (1:1000), p-MLKL (1:1000), p-JAK2 (1:1000), JAK2 (1:1000), p-STAT3 (1:1000), STAT3 (1:2000), SIRT6 (1:1000), and GAPDH (1:1000) overnight at 4 °C and HRP-linked secondary antibodies for 1 hr at RT. Bands were detected using enhanced chemiluminescence (ECL) prime reagent. The intensity of bands was assessed using ImageJ software.

***Statistics***

The results are expressed as the mean ± SD. The statistical significance of the experimental data was analyzed by one-way ANOVA. *P*<0.05 was considered to indicate statistical significance.

## Results

***Vitexin improves the survival of GCDC-induced THLE-3 cells and decreases apoptosis***

**Figure 1:**
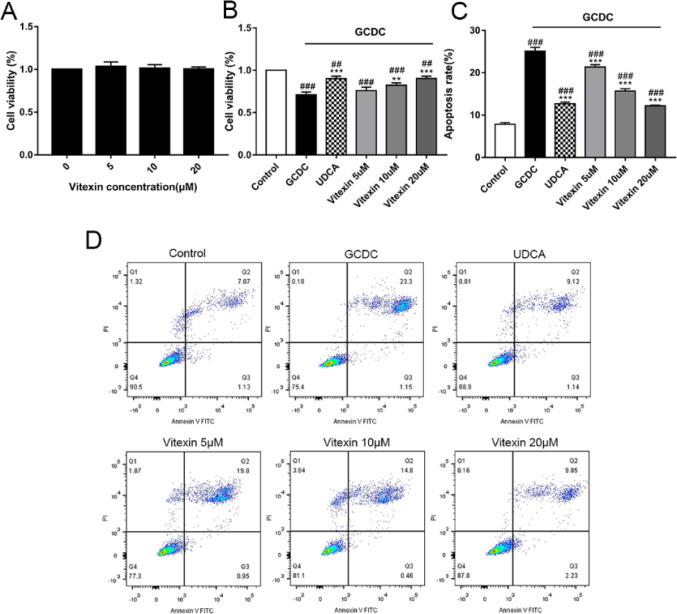
(A) Effects of vitexin on THLE-3 cell viability. (B) Effects of vitexin on GCDC-treated THLE-3 cell viability. (C) (D) Inhibitory apoptosis effects of vitexin on GCDC-treated THLE-3 cells. #*P*<0.05, ## *P*<0.01, and ### *P*<0.001 vs the control groups; *P<0.05, ** *P*<0.01, and *** *P*<0.001 vs the GCDC groups. Values are the means ± SD for all groups, n=3

To evaluate the cytoprotective effect of vitexin on GCDC-treated THLE-3 cells, CCK-8 assays were carried out. First, we found that vitexin had no cytotoxicity on THLE-3 cells in the treatment range (5 μM, 10 μM, and 20 μM) (*P*>0.05) (Figure 1A).  In contrast, the viability of GCDC-treated THLE-3 cells was significantly less than that of the controls (*P*<0.001). Vitexin increased the viability of GCDC-treated THLE-3 cells in a dose-dependent manner (*P*<0.001 or *P*<0.01), and 20 μM vitexin was chosen for further study (Figure 1B).

Through FCM, we further measured cell apoptosis. The results showed that the GCDC-induced increase in the apoptosis rate was inhibited by increasing concentrations of vitexin (*P*<0.001) (Figures 1C, D). These results suggest that vitexin exerts a dose-dependent protective effect on GCDC-induced THLE-3 cell injury by promoting cell viability and suppressing apoptosis.

***Vitexin reduces oxidative stress in GCDC-treated THLE-3 cells***

**Figure 2:**
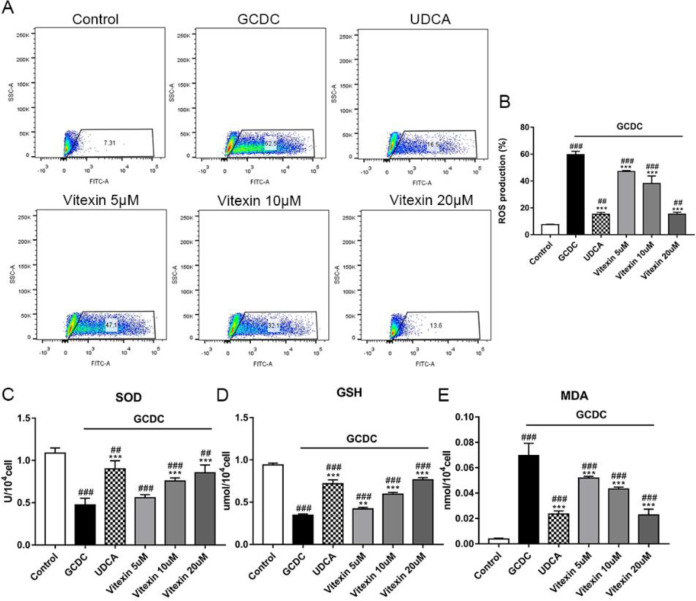
Effects of vitexin on the production of (A) (B) ROS, (C-E) SOD, GSH, and MDA in GCDC-induced THLE-3 cells. #*P*<0.05, ## *P*<0.01, and ### *P*<0.001 vs the control groups; *P<0.05, ** *P*<0.01, and *** *P*<0.001 vs the GCDC groups. Values are the means ± SD for all groups, n=3

Intracellular ROS production increased notably in GCDC-exposed cells (*P*<0.001), and preincubation with various concentrations of vitexin caused a significant decrease in GCDC-induced ROS generation (*P*<0.001) (Figures 2A, B).

Additionally, oxidative stress was assessed by detecting MDA levels and SOD and GSH activities. As shown in Figures 2C-E, GCDC induced an elevation in MDA content and depressed SOD and GSH activities in THLE-3 cells (*P*<0.001). Vitexin treatment markedly reduced MDA production and elevated SOD and GSH activities (*P*<0.001 or *P*<0.01).

***Vitexin increases MMP in GCDC-treated THLE-3 cells***

**Figure 3:**
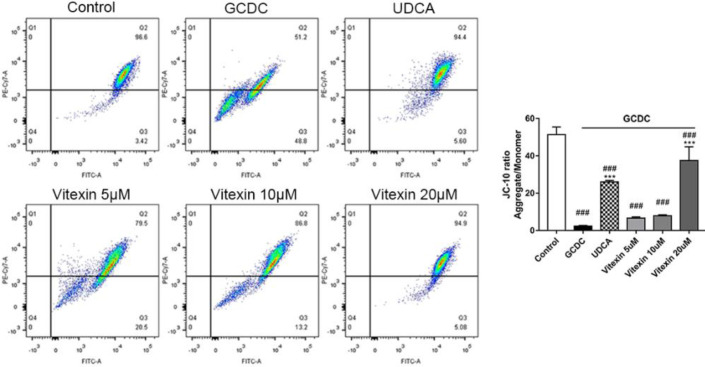
Effects of vitexin on MMP in GCDC-treated THLE-3 cells. #*P*<0.05, ## *P*<0.01, and ### *P*<0.001 vs the control groups; *P<0.05, **P<0.01, and ***P<0.001 vs the GCDC groups. Values are means ± SD for all groups, n=3

JC-10 assay was performed to evaluate MMP. JC-10, a derivative of JC-1, is an ideal fluorescent probe extensively used to determine MMP. JC-10 aggregates (red fluorescence) in the mitochondria of normal cells and forms a monomer (green fluorescence) under depolarizing conditions. We noticed that cells treated with GCDC for 24 hr exhibited a significant breakdown of MMP (*P*<0.001). Treatment with vitexin significantly ameliorated this decrease in a dose-dependent manner (*P*<0.001) (Figure 3).

***Vitexin restrains GCDC-induced THLE-3 cell necroptosis***

**Figure 4:**
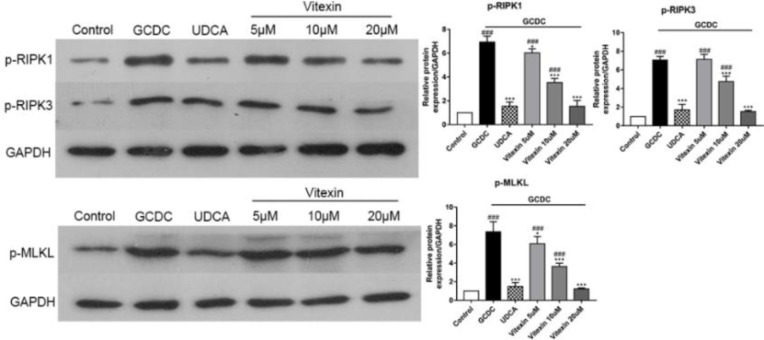
Influences of vitexin on necroptosis-associated factors (p-RIPK1, p-RIPK3, and p-MLKL) in GCDC-treated THLE-3 cells. #*P*<0.05, ## *P*<0.01, and ### *P*<0.001 vs the control groups; *P<0.05, ** *P*<0.01, and *** *P*<0.001 vs the GCDC groups. Values are the means ± SD for all groups, n=5

Next, we examined the expression of the major necroptosis factors RIPK1, RIPK3, and MLKL in GCDC-treated THLE-3 cells. Cell lysates were harvested and subjected to Western blot analysis for phosphorylation of RIPK1, RIPK3, and MLKL. Our observations suggested that RIPK1, RIPK3, and MLKL were phosphorylated upon GCDC stimulation (*P*<0.001), while the addition of vitexin attenuated the phosphorylation of RIPK1, RIPK3, and MLKL (*P*<0.001 or *P*<0.05) (Figure 4).

***Total anti-oxidant capacity of vitexin is reversed*** *by SIRT6 inhibitor*

**Figure 5:**
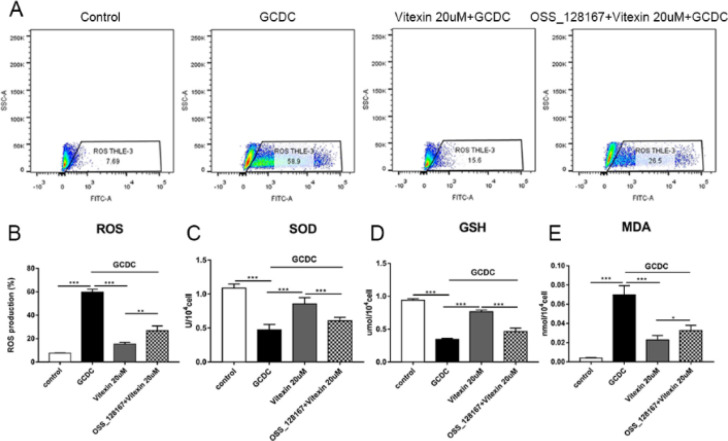
SIRT6 inhibitor OSS_128167 reversed the total anti-oxidant capacity of vitexin. *P<0.05, **P<0.01, and ***P<0.001. Values are the means ± SD for all groups, n=3

To explore the underlying anti-oxidant mechanism of vitexin, we added the SIRT6 inhibitor OSS_128167 before the addition of vitexin. The results showed that, compared to that in the vitexin treatment group, ROS MDA production increased and SOD GSH activities decreased after addition of the SIRT6 inhibitor (*P*<0.001, *P*<0.01, or *P*<0.05) (Figure 5).

***Antiapoptotic*** ***ability*** ***of vitexin is inhibited*** ***by*** ***a SIRT6 inhibitor***

**Figure 6:**
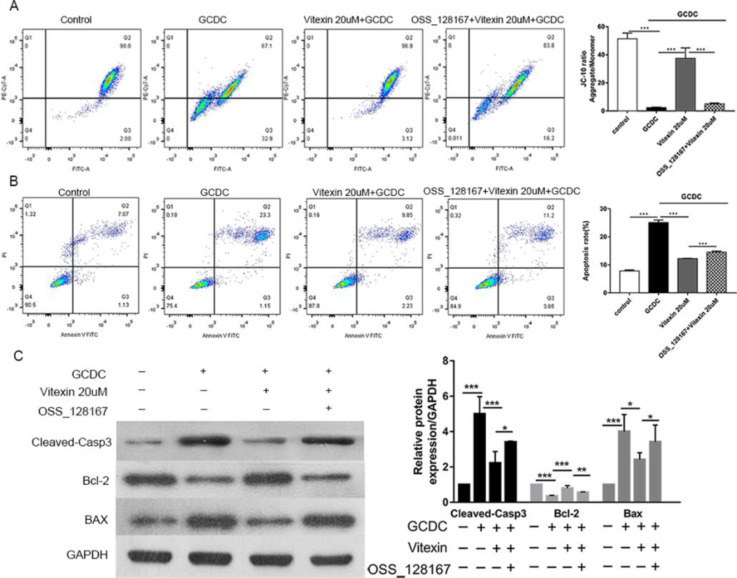
The SIRT6 inhibitor OSS_128167 inhibited the total antiapoptotic capacity of vitexin. (A) The SIRT6 inhibitor OSS_128167 reversed the ability of vitexin to inhibit the decrease in MMP caused by GCDC. (B) The SIRT6 inhibitor OSS_128167 reversed the capacity of vitexin to inhibit GCDC-induced apoptosis. (C) Influence of the SIRT6 inhibitor OSS_128167 on the protein levels of apoptosis-associated indicators (cleaved casp3, Bax, and Bcl-2). *P<0.05, **P<0.01, and ***P<0.001. Values are the means ± SD for all groups, n=5

To explore the underlying antiapoptotic mechanism of vitexin, we added the SIRT6 inhibitor OSS_128167 before the addition of vitexin. First, reduction of MMP is a hallmark of the early apoptotic process. We found that OSS_128167 partially reversed the ability of vitexin to inhibit the decrease in MMP caused by GCDC (*P*<0.001) (Figure 6A). FCM results showed that OSS_128167 also reversed the capacity of vitexin to inhibit GCDC-induced apoptosis (*P*<0.001) (Figure 6B).

Moreover, at the molecular level, we further investigated the protein levels of apoptosis-associated indicators (cleaved casp3, Bax, and Bcl-2) by Western blot. After adding OSS_128167, cleaved casp3 and Bax expression were higher than that in the vitexin treatment group (*P*<0.05). The expression pattern of Bcl-2 was opposite to that of cleaved casp3 and Bax (*P*<0.01) (Figure 6C).

***The anti-necroptotic*** ***ability of vitexin is blocked by a SIRT6 inhibitor***

**Figure 7:**
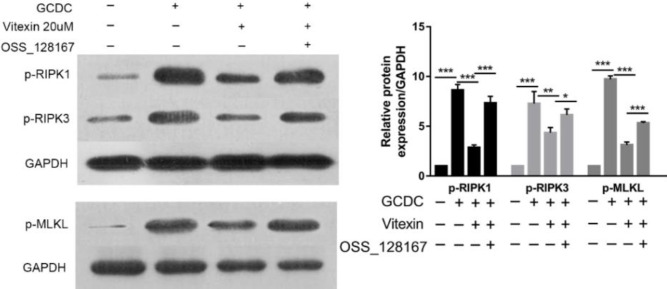
SIRT6 inhibitor OSS_128167 blocked the total anti-necroptotic ability of vitexin. *P*<0.05, **P<0.01, and ***P<0.001. Values are the means ± SD for all groups, n=5

To explore the underlying anti-necroptotic mechanism of vitexin, we added the SIRT6 inhibitor OSS_128167 before addition of vitexin. The results indicated that compared with that in the vitexin treatment group, expression of p-RIPK1, p-RIPK3, and p-MLKI was increased after the addition of OSS_128167 (*P*<0.001 or *P*<0.05) (Figure 7).

***Vitexin can inhibit the JAK2/STAT3 pathway by promoting SIRT6 expression***

**Figure 8:**
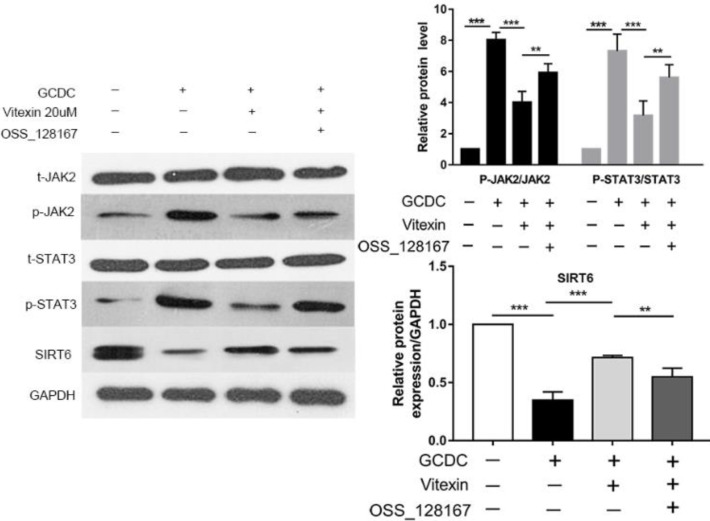
Effects of vitexin on the protein expression of SIRT6 and the JAK2/STAT3 pathways in GCDC-treated THLE-3 cells. *P*<0.05, **P<0.01, and ***P<0.001. Values are the means ± SD for all groups, n=5

To investigate the relationship between vitexin-induced SIRT6 and JAK2/STAT3 cascade expression, we assessed the effects of the SIRT6 inhibitor OSS_128167 on JAK2/STAT3 activation. When THLE-3 cells were exposed to GCDC, the protein expression of SIRT6 was decreased and p-JAK2 and p-STAT3 levels were markedly increased (*P*<0.001), and vitexin treatment further up-regulated SIRT6 protein expression and down-regulated p-JAK2 and p-STAT3 protein expression compared with that in the GCDC group (*P*<0.001). Compared with vitexin treatment, OSS_128167 markedly decreased the protein expression of SIRT6 (*P*<0.01), demonstrating that the inhibitor was effective. In addition, the phosphorylation of JAK2 and STAT3 was up-regulated by OSS_128167 (*P*<0.01) (Figure 8).

## Discussion

Cholestasis is characterized by impaired bile flow, resulting in the accumulation of BAs in hepatocytes and serum. The toxicity of BAs can cause hepatocellular injury, followed by inflammation, hepatic fibrosis, and cirrhosis, leading to portal hypertension and hepatic failure. Since current drug therapy is limited by poor efficacy, patients with end-stage cholestasis usually require liver transplantation (23, 24). Hepatocytes are constantly exposed to high concentrations of hydrophobic BAs in CLDs. GCDC is one of the most toxic hydrophobic BAs (25). The pathogenesis of GCDC-induced hepatocyte injury remains elusive, Thus, further investigations to elucidate the mechanisms responsible for BA-induced hepatocyte injury should be performed (26).

Dysregulation of cell death is closely related to the pathogenesis of CLDs (27). Programmed cell death plays an important role in regulating the pathogenesis of CLDs, especially necroptosis and apoptosis, which are mediated by the receptor-interacting serine/threonine-protein kinase (RIPK)1/3 and caspase-3 signaling pathways, respectively (28). Both necroptosis and apoptosis are likely to be present in cholestatic diseases, it depends on the length, cause, and complications of the disease (7).

In-depth studies on the regulatory mechanism of apoptosis find that an abnormal increase in apoptosis is an important factor in the pathogenesis of some hepatobiliary diseases. Apoptosis is considered a vital component of various biological processes and a typical feature of various hepatic diseases. Pathological changes associated with cholestasis usually include hepatocyte shrinkage and formation of acidophilic bodies, which are manifestations of apoptosis (29, 30). Previous studies have noted that hepatocyte apoptosis occurs in mice after bile duct ligation (BDL). Recent reports suggest that bile acids can lead to hepatocyte apoptosis in cholestatic jaundice (3, 31). In this study, hepatocyte viability and apoptosis were detected using CCK-8 assay and FCM. In addition, the effect of vitexin on GCDC-induced hepatocyte injury was evaluated. The results showed that vitexin protected hepatocytes from GCDC-induced injury by promoting viability and inhibiting apoptosis in a dose-dependent manner, while the SIRT6 inhibitor OSS_128167 significantly weakened the inhibitory effect of vitexin on apoptosis. This indicates that SIRT6 plays a role in vitexin’s regulation of apoptosis.

An increasing number of studies suggest that cell death in liver diseases is associated with necroptosis (32). Findings based on BDL of the mouse models suggested a positive correlation between the expression of RIPK3 and MLKL and liver injury; moreover, knockout of RIPK3 could improve cholestasis-related inflammatory necrosis, which was especially effective soon after BDL (33). However, it remains unclear whether necroptosis is vital to the pathogenesis of CLDs. Moreover, no study has yet investigated whether GCDC can induce hepatocyte necrosis. The results of the present study found that vitexin inhibited the expression of p-RIPK1, p-RIPK3, and p-MLKL in GCDC-treated hepatocytes, confirming that vitexin protected hepatocytes against GCDC-induced necroptosis. While the SIRT6 inhibitor OSS_128167 partially inhibited the protective effect of vitexin, suggesting that vitexin regulates GCDC-induced hepatocyte necroptosis via a SIRT6-associated pathway.

Both necrosis and apoptosis involve the mitochondria. Increasing evidence suggests that BAs can damage mitochondrial function in hepatocytes by inhibiting the activity of the mitochondrial respiratory chain complex and increasing the production of reactive oxygen species (ROS) (6, 7). GCDC is a well-known mitochondrial toxin (19). Mitochondria are high-energy organelles that produce ATP through oxidative phosphorylation based on electron transfer via the respiratory chain. When mitochondrial dysfunction occurs, electrons overflow from the respiratory chain and react with oxygen and produce ROS, which disrupts redox homeostasis. Moreover, subsequent oxidative stress can exogenously induce hepatotoxicity (6, 34). The results of the present study revealed that, compared with the control group, ROS and MDA levels were increased in the GCDC group, while SOD and GSH levels were decreased. These indicated that GCDC causes oxidative damage to hepatocytes. The addition of vitexin to the GCDC group decreased ROS and MDA levels and increased SOD and GSH levels, suggesting that vitexin has protective effects on oxidative damage to hepatocytes. The anti-oxidant activity of vitexin was significantly impaired by the SIRT6 inhibitor OSS_128167, suggesting that vitexin regulates the anti-oxidant capacity of hepatocytes through the SIRT6 pathway.

Once ROS decreases MMP and increases mitochondrial membrane permeability, mitochondrial proapoptotic factors will be released to the cytoplasm and trigger the apoptotic process via activation of the caspase cascade (34). Depolarization of MMP occurs in the early stages of apoptosis (35). In the present study, the cationic dye JC-10 was used to analyze the integrity of the mitochondrial membrane. The results showed that the expression levels of MMP were significantly decreased in GCDC-treated THLE-3 cells, while vitexin reversed this trend in a dose-dependent manner, indicating that vitexin may regulate the expression levels of MMP via SIRT6. In CLDs, mitochondrial dysfunction and oxidative stress-induced cell death. Therefore, preventing oxidative stress and mitochondrial dysfunction of hepatocytes can improve hepatic function and ameliorate cholestasis (6).

It is reported that SIRT6 relieves liver injury and fibrosis in BDL mouse models by inhibiting the expression of estrogen-related receptor γ (ERRγ)(11). Studies also show that SIRT6 up-regulates the expression of the Peroxisome proliferator-activated receptor gamma coactivator 1-alpha (PGC-1α) and promotes deacetylation via the AMP-activated protein kinase (AMPK) pathway to improve GCDC-induced apoptosis of HiBEC cells (6). These findings suggest that activated SIRT6 may represent a promising novel treatment option for cholestatic liver injury. Zhong *et al*. found that protein expression of p-JAK2 and p-STAT3 is markedly enhanced in the hepatic ischemia-reperfusion injury tissues, and JAK2/STAT3 pathway promotes hepatocellular cell death (36). In the fibrotic liver, JAK2/STAT3 pathway is also able to influence liver fibrosis (16). In our study, we found that GCDC can down-regulate SIRT6 expression and enhance the JAK2/STAT3 pathway. And vitexin significantly up-regulated SIRT6 expression and suppress the JAK2/STAT3 pathway. When OSS_128167, a SIRT6 inhibitor was added to cells, the inhibitory effect of vitexin on the JAK2/STAT3 pathway was attenuated, and the inhibitory effects of vitexin on oxidative stress, apoptosis, and necroptosis were also significantly weakened. This illustrated vitexin is an agonist of SIRT6, which can alleviate GCDC-induced THLE-3 cell injury by up-regulating SIRT6 expression and inhibiting the JAK2/STAT3 pathway.

## Conclusion

According to the results of this study, GCDC triggered THLE-3 cell apoptosis and necroptosis, declined MMP, and stimulated oxidative stress by reducing SIRT6 expression and enhancing the JAK2/STAT3 pathway. Vitexin ameliorated GCDC-induced THLE-3 cell injury by activating SIRT6 and suppressing the JAK2/STAT3 pathway. As a promising medication for cholestatic hepatocellular injury, vitexin provides a novel treatment option for patients with CLDs.

## Authors’ Contributions

SL and CZ designed the experiments; CZ, XP, and BZ performed experiments and collected data; CZ, CS, and LL discussed the results and strategy; CZ, YF, and XY prepared and visualized the draft manuscript; SL critically revised the article; SL, YF, and XY supervised, directed, and managed the study; SL, CS, and LL approved the final version to be published.

## Conflicts of Interest

No potential conflicts of interest was reported by the authors.
